# High-dose intensity-modulated radiotherapy for prostate cancer using daily fiducial marker-based position verification: acute and late toxicity in 331 patients

**DOI:** 10.1186/1748-717X-3-15

**Published:** 2008-05-21

**Authors:** Irene M Lips, Homan Dehnad, Carla H van Gils, Arto E Boeken Kruger, Uulke A van der Heide, Marco van Vulpen

**Affiliations:** 1 Department of Radiation Oncology, University Medical Center Utrecht, The Netherlands; 2 Julius Center for Health Sciences and Primary Care, University Medical Center Utrecht, The Netherlands; 3 Department of Urology, University Medical Center Utrecht, The Netherlands

## Abstract

We evaluated the acute and late toxicity after high-dose intensity-modulated radiotherapy (IMRT) with fiducial marker-based position verification for prostate cancer. Between 2001 and 2004, 331 patients with prostate cancer received 76 Gy in 35 fractions using IMRT combined with fiducial marker-based position verification. The symptoms before treatment (pre-treatment) and weekly during treatment (acute toxicity) were scored using the Common Toxicity Criteria (CTC). The goal was to score late toxicity according to the Radiation Therapy Oncology Group/European Organization for Research and Treatment of Cancer (RTOG/EORTC) scale with a follow-up time of at least three years. Twenty-two percent of the patients experienced pre-treatment grade ≥ 2 genitourinary (GU) complaints and 2% experienced grade 2 gastrointestinal (GI) complaints. Acute grade 2 GU and GI toxicity occurred in 47% and 30%, respectively. Only 3% of the patients developed acute grade 3 GU and no grade ≥ 3 GI toxicity occurred. After a mean follow-up time of 47 months with a minimum of 31 months for all patients, the incidence of late grade 2 GU and GI toxicity was 21% and 9%, respectively. Grade ≥ 3 GU and GI toxicity rates were 4% and 1%, respectively, including one patient with a rectal fistula and one patient with a severe hemorrhagic cystitis (both grade 4). In conclusion, high-dose intensity-modulated radiotherapy with fiducial marker-based position verification is well tolerated. The low grade ≥ 3 toxicity allows further dose escalation if the same dose constraints for the organs at risk will be used.

## Findings

Several randomized trials have demonstrated a significant benefit of an increased radiation dose for the treatment of prostate cancer [1-3]. Further dose escalation is expected to lead to further improvement [4]. However, dose escalation is associated with an increased risk of acute and late toxicity [1-3].

Prostate tumour cells are predominantly located in the peripheral zone of the prostate situated at the dorsal site [5]. Therefore, the challenge is to achieve a sufficiently high-dose to the peripheral zone of the prostate, while providing an adequate sparing of the rectum. Intensity-modulated radiotherapy (IMRT) is able to deliver such dose distributions and has therefore become the preferred treatment technique [6-11].

Sharp dose gradients between the target volume and the organ at risk require reliable and accurate position verification to prevent decreased biochemical control and increased rectal toxicity [12]. Fiducial gold markers implanted in the prostate have proved to be reliable markers of prostate position over the course of radiation treatment [13]. Their position can be easily and automatically detected with electronic portal imaging devices, allowing for fast and accurate determination of the prostate position. Daily correction of the position of the prostate using fiducial markers minimizes the setup uncertainties [14].

Several prospective and randomized trials have accurately presented the incidences of their acute and late toxicity [3,7-9,15-18]. Only Skala et al. [9] reported toxicity rates after prostate cancer treatment with three-dimensional (3D) conformal/IMRT using fiducial marker-based position verification. They collected patient-reported questionnaires of 365 patients to determine the incidence of late toxicity. Until now, no longitudinal study of physician-reported toxicity including baseline measurements has been published for patients treated with IMRT using fiducial markers. Therefore, we describe in this study the complete pre-treatment symptoms and the acute and late toxicity of a large number of patients treated with high-dose IMRT using daily fiducial marker-based position verification.

According to literature, a follow-up of three years is sufficient for the majority of later rectal morbidity to manifest itself [2,3]. Therefore, we evaluated toxicity in the entire population of patients (n = 331) treated at our department from August 2001 until December 2004, which resulted in a minimum follow-up time of 31 months for all patients. The prostate was delineated on a CT-scan and a margin of 8 mm was applied to the prostate and seminal vesicles to create a planning target volume (PTV). Patients received an IMRT treatment using a five-beam step-and-shoot technique [14,19]. A mean dose of 76 Gy in 35 fractions was prescribed to the PTV and 95% of the prescription dose (= 72 Gy) was prescribed to 99% of the PTV. The dose to the overlapping region with rectum and bladder was limited so that no more than 5% of the rectum and 10% of the bladder would receive a dose of ≥ 72 Gy [20]. No elective pelvic node irradiation was performed.

Fiducial markers for position verification were transrectally implanted with the use of antibiotic prophylaxis [13]. Daily portal images of the fiducial markers were taken to determine the position variations during treatment. With the use of an offline adapted shrinking action level (SAL) protocol the systematic errors were less than 0.8 mm in all directions [14].

Pre-treatment symptoms and acute toxicity were scored using the Common Toxicity Criteria (CTC) version 2.0 [21]. Acute toxicity was present when one of the symptoms occurred within 90 days after the start of treatment [21]. Late toxicity was scored according to the Radiation Therapy Oncology Group/European Organization for Research and Treatment of Cancer (RTOG/EORTC) morbidity scale version 9 [22], because the CTC version 2.0 only focuses on acute effects [21]. Follow-up took place 4 weeks after treatment, every 3 months in the first year and every 6 months thereafter at the department of radiotherapy. Every symptom was counted even if it occurred only on one single occasion.

The patient characteristics of the 331 patients are presented in Table 1. The mean follow-up time was 47 months (range: 31–71 months). At the time of study entry, no national guidelines for hormonal treatment were available. Therefore, only 95 patients received adjuvant hormonal treatment. Bone scan and/or pelvic lymph node dissection was performed in all patients with PSA levels above 20 ng/ml to rule out M+ disease. Late side effects with a minimum follow-up time of 31 months were available for 320 patients, because three patients died and eight patients were lost to follow-up during the first three years.

**Table 1:** Patient characteristics of the 331 patients

Characteristics	
Age at baseline (year), Mean (range)	68 (46 – 80)
Initial PSA value (ng/mL), Mean (range)	20 (0.5 – 175)
	
Biopsy Gleason score	
≤ 4	39 (12)
5 – 7	228 (69)
≥ 8	64 (19)
	
Tumor stage	
T1	37 (11)
T2	31 (9)
T3	262 (79)
T4	1 (1)
	
Hormonal treatment	
None	236 (71)
Short-term	70 (21)
Long-term	25 (8)
TURP	40 (12)

*Abbreviations*: TURP = transurethral resection of the prostate.

PSA = prostate specific antigen.

Values are number (percentage), unless otherwise noted.

In Table 2, the grades of pre-treatment symptoms and acute and late toxicity are shown. The highest toxicity score for each patient was used, to calculate an overall GU and GI score of the CTC items. Seventy-three patients (22%) showed pre-treatment GU symptoms of grade ≥ 2 and six patients (2%) experienced grade 2 proctitis complaints before radiotherapy.

**Table 2:** Pre-treatment complaints and acute toxicity according to the Common Toxicity Criteria (CTC) and late toxicity according to the Radiation Therapy Oncology Group/European Organization for Research and Treatment of Cancer (RTOG/EORTC) scale

Item	Number of patients (%)				
	
	Grade 0	Grade 1	Grade 2	Grade 3	Grade 4
*Pre-treatment (n = 331)*					
Genitourinary					
Urinary frequency/urgency	161 (49)	99 (30)	69 (21)	2 (1)	0 (0)
Urinary retention	317 (96)	13 (4)	1 (0.3)	0 (0)	0 (0)
Bladder spasms	328 (99)	3 (1)	0 (0)	0 (0)	0 (0)
Urinary incontinence	318 (96)	13 (4)	0 (0)	0 (0)	0 (0)
Hematuria	324 (98)	6 (2)	1 (0.3)	0 (0)	0 (0)
Dysuria	318 (96)	13 (4)	0 (0)	0 (0)	0 (0)
Overall	150 (45)	108 (33)	71 (22)	2 (1)	0 (0)
					
Gastrointestinal					
Proctitis	306 (92)	19 (6)	6 (2)	0 (0)	0 (0)
Rectal or perirectal pain	328 (99)	3 (1)	0 (0)	0 (0)	0 (0)
Overall	305 (92)	20 (6)	6 (2)	0 (0)	0 (0)
					
*Acute toxicity (n = 331)*					
Genitourinary					
Urinary frequency/urgency	25 (8)	154 (47)	144 (44)	8 (2)	0 (0)
Urinary retention	271 (82)	52 (16)	3 (1)	5 (2)	0 (0)
Bladder spasms	309 (94)	18 (5)	4 (1)	0 (0)	0 (0)
Urinary incontinence	305 (92)	23 (7)	3 (1)	0 (0)	0 (0)
Hematuria	317 (96)	7 (2)	6 (2)	1 (0.3)	0 (0)
Dysuria	165 (50)	139 (42)	26 (8)	1 (0.3)	0 (0)
Overall	19 (6)	147 (44)	155 (47)	10 (3)	0 (0)
					
Gastrointestinal					
Proctitis	71 (22)	168 (51)	92 (28)	0 (0)	0 (0)
Rectal or perirectal pain	275 (83)	32 (10)	24 (7)	0 (0)	0 (0)
Overall	63 (19)	169 (51)	99 (30)	0 (0)	0 (0)
					
Infection	313 (95)	3 (1)	12 (4)	3 (1)	0 (0)
					
*Late toxicity (n = 320)*					
Genitourinary	152 (48)	86 (27)	68 (21)	13 (4)	1 (0.3)
Gastrointestinal	193 (60)	94 (29)	30 (9)	2 (1)	1 (0.3)

Acute grade 2 GU and GI toxicity was found in 47% and 30% of our patient group. Ten patients (3%) developed grade 3 acute GU side effects with two patients having a urinary catheter before treatment (grade 3) and six patients having pre-treatment grade 2 GU symptoms. Acute grade 3 infections were seen in three patients: respectively a urinary tract infection, a pneumonitis and a prostatitis after marker implantation, that all needed intravenous antibiotic. No grade 4 acute toxicity was seen for both GU and GI. Ninety-nine percent of the patients with pre-treatment grade ≥ 2 GU symptoms demonstrated acute grade ≥ 2 toxicity, compared to 36% of the patients with pre-treatment GU complaints of < grade 2. As grade 3 toxicity seldom occurred, most patients with pretreatment grade 2 complaints mainly continued having grade 2 toxicity during treatment.

Eighty-two and 33 patients demonstrated late grade ≥ 2 GU and GI toxicity, respectively. Two patients experienced late grade 4 morbidities: one patient experienced a severe haemorrhagic cystitis and required a suprapubic catheter. Three months before the start of the radiotherapy he underwent a TURP and he had pre-treatment grade 1 urinary frequency/urgency complaints and acute grade 1 dysuria and grade 2 hematuria and urinary frequency/urgency toxicity. Furthermore, this patient suffered from late grade 2 GI toxicity with frequent bleeding that required steroid enemas. The other patient developed a rectal fistula requiring surgery 18 months after radiotherapy. This patient had no pre-treatment GI complaints, but during radiotherapy he developed grade 2 perirectal pain and proctitis. For both patients the technical and dosimetric details of their radiotherapy treatment were evaluated and no abnormalities were found.

The incidence of late grade ≥ 2 GU toxicity for patients with pre-treatment grade ≥ 2 GU complaints was 58%, compared to 17% for patients with grade < 2 GU symptoms before radiotherapy. Calculation of relative risks (RR) accompanying 95% confidence intervals (95%-CI) demonstrated for patients with acute grade ≥ 2 GU complaints a 5.2 fold (95%-CI: 3.0–9.1) increased risk for late grade ≥ 2 GU compared to those who had acute grade < 2 GU complaints. Additionally, the risk of late grade ≥ 2 GI toxicity was increased for patients with acute grade ≥ 2 GI complaints (RR: 2.2; 95%-CI: 1.1–4.1).

This data demonstrates that a dose of 76 Gy in 35 fractions, using IMRT and daily fiducial marker-based position verification, is well tolerated. Acute and late toxicity from different studies, when available, are presented in Table 3. The acute toxicity established in our patient group, in particular grade ≥ 3, was lower than reported in literature for 3D conformal radiotherapy [3,15-18]. Although different toxicity scales and radiotherapy techniques make a comparison difficult. De Meerleer et al. [7] treated 114 patients with high-dose IMRT with position verification by visualizing the bony anatomy and reported comparable acute GI toxicity rates and somewhat lower grade 2 and higher grade 3 acute GU toxicity rates. Zelefsky et al. [8] reported lower acute toxicity rates after high-dose IMRT with lower fraction doses of only 1.8 Gy. As in most other toxicity reports acute GU toxicity was more pronounced than GI toxicity [7,8,15,17,18].

**Table 3:** Acute and late toxicity from different studies

Authors	Acute toxicity						Late toxicity					
		
	GU (%)			GI (%)			GU (%)			GI (%)		
	Grade			Grade			Grade			Grade		
	2	3	4	2	3	4	2	3	4	2	3	4

*3D-conformal radiotherapy*												
Storey, 2000 [18], Pollack 2002 [2]	24	4	1	43	0	0	10	3	-	19	7	-
Beckendorf, 2004 [15]	30	7	-	28	2	-	-	-	-	-	-	-
Michalski, 2005 [16]	41	3	0	41	3	0	17	4	0	18	2	1
Zietman, 2005 [3]	49	1	1	57	0	0	20	1	0	17	1	0
Peeters, 2005/2006 [1,17]	42	13	0	47	4	0	26	13	-	27	5	-
												
*Intensity-modulated radiotherapy*												
Zelefsky, 2002/2006 [8,11]	28	0.1	0	5	0	0	9	3	0	2	0.1	0
De Meerleer, 2004/2007 [7,10]	36	7	0	29	0	0	19	3	0	17	1	0
Teh, 2005 [23]	35	0	0	6	0	0	-	-	-	7	2	0
Skala, 2007 [9]	-	-	-	-	-	-	9	1	-	3	1	-	
Current study	47	3	0	30	0	0	21	4	0.3	9	1	0.3

*Abbreviations*: GU = genitourinary; GI = gastrointestinal; - = toxicity rate not available.

The randomized dose-escalation trials reported more late GI and comparable late GU morbidities [2,3,17]. One hundred sixteen patients, treated with IMRT using a rectal balloon for position verification, demonstrated comparable late GI toxicity [23]. De Meerleer et al. [10] reported slightly higher late GI toxicity and comparable GU toxicity rates for 133 patients treated with IMRT. Zelefsky et al. [11] described lower incidences of late toxicity for IMRT after a median follow-up time of only 24 months. Skala et al. [9] reported somewhat lower late GU and GI toxicity rates, however the cross-sectional toxicity data was collected from patient-reported questionnaires.

Patients with pre-treatment grade 2 complaints mainly remained acute and late grade 2 toxicity. The predictive value of pre-treatment symptoms has also been reported by others [17,24-26].

Although our patients had a median follow-up time of 47 months and all patients had a follow-up time of at least 31 months, continuing scoring of toxicity is needed, because an increase in GU complications has been reported after three years [27].

In conclusion, a dose of 76 Gy in 35 fractions using IMRT and fiducial marker-based position verifications is well tolerated, because the low incidences of grade ≥ 3 acute and late GU and GI side effects. These results provide possibilities for further dose escalation, because acceptable toxicity is expected when the same dose constraints for the organs at risk and good quality external beam radiotherapy are being used.

## Competing interests

The authors declare that they have no competing interests.

## Authors' contributions

IL participated in data collection and drafted the manuscript. HD participated in data collection. CG participated in data analysis. ABK participated in the design of the study. UH revised the manuscript critically. MV participated in its design and coordination and helped to draft the manuscript. All authors read and approved the final manuscript.
